# Efficacy and Safety of Garadacimab in Combination with Standard of Care Treatment in Patients with Severe COVID-19

**DOI:** 10.1007/s00408-023-00615-9

**Published:** 2023-03-31

**Authors:** Alberto Papi, Renee D. Stapleton, Paul M. Shore, Mihai Alexandru Bica, Younan Chen, Michael Larbig, Tobias Welte

**Affiliations:** 1 Respiratory Department, University of Ferrara, 44100 Ferrara, Italy; 2 Pulmonary and Critical Care Medicine, University of Vermont, Burlington, VT USA; 3 Clinical Development, CSL Behring, King of Prussia, USA; 4 Global Clinical Safety and Pharmacovigilance, CSL Behring Innovation, Marburg, Germany; 5 Biostatistics, CSL Behring, King of Prussia, USA; 6 Clinical Development, CSL Behring AG, Bern, Switzerland; 7 Department of Pulmonary and Infectious Diseases, Hannover Medical School, Hannover, Germany

**Keywords:** COVID-19, Critically ill patients, Inflammation, Respiratory disease

## Abstract

**Background:**

Garadacimab, a fully human IgG4 monoclonal antibody, inhibits the kallikrein–kinin pathway at a key initiator, activated coagulation factor XII (FXIIa), and may play a protective role in preventing the progression of COVID-19. This phase 2 study evaluated the efficacy and safety of garadacimab plus standard of care (SOC) versus placebo plus SOC in patients with severe COVID-19.

**Methods:**

Patients hospitalised with COVID-19 were randomised (1:1) to a single intravenous dose of garadacimab (700 mg) plus SOC or placebo plus SOC. Co-primary endpoint was incidence of endotracheal intubation or death between randomisation and Day 28. All-cause mortality, safety and pharmacokinetic/pharmacodynamic parameters were assessed.

**Results:**

No difference in incidence of tracheal intubation or death (*p* = 0.274) or all-cause mortality was observed (*p* = 0.382). Garadacimab was associated with a lower incidence of treatment-emergent adverse events (60.3% vs 67.8%) and fewer serious adverse events (34 vs 45 events) versus placebo. No garadacimab-related deaths or bleeding events were reported, including in the 45.9% (*n* = 28/61) of patients who received concomitant heparin. Prolonged activated partial thromboplastin time (aPTT), and increased coagulation factor XII (FXII) levels were observed with garadacimab versus placebo to Day 14, whilst FXIIa-mediated kallikrein activity (FXIIa-mKA) was suppressed to Day 28.

**Conclusion:**

In patients with severe COVID-19, garadacimab did not confer a clinical benefit over placebo. Transient aPTT prolongation and suppressed FXIIa-mKA showed target engagement of garadacimab that was not associated with bleeding events even with concomitant anticoagulant use. The safety profile of garadacimab was consistent with previous studies in patients with hereditary angioedema.

**ClinicalTrials. gov Identifier:**

NCT04409509. Date of registration: 28 May, 2020.

**Supplementary Information:**

The online version contains supplementary material available at 10.1007/s00408-023-00615-9.

## Introduction

Coronavirus disease 2019 (COVID-19) is an infectious disease caused by severe acute respiratory syndrome coronavirus 2 (SARS-CoV-2). Although many patients with symptomatic COVID-19 experience mild illness, data from China report ~ 14% of unvaccinated patients with COVID-19 develop severe disease and 5% develop critical illness [1] requiring mechanical ventilation and admission to intensive care [2, 3].

Pathophysiological mechanisms underlying disease severity in COVID-19 remain unclear, although dysregulated immune responses have been implicated in progression of infection to severe disease [4, 5]. Numerous potential therapeutic options, including immune modulation and adjuvant antiviral therapies, are under investigation for treating COVID-19-related respiratory disease; many have shown clinical benefit [6, 7]. mRNA COVID-19 vaccines have proven highly effective at preventing hospital admissions for all three identified COVID-19 variants and are associated with reduced progression to severe disease [8].

The functional ligand and entry receptor of SARS-CoV-2 is human angiotensin-converting enzyme (ACE-2) [9, 10]. Host binding of SARS-CoV-2 to ACE-2 has been reported to impair hydrolysis of des-Arg9 bradykinin (BK) [11] through increased activation of BK receptors 1 and 2, resulting in vascular leakage promoting pulmonary oedema [11–13]—major contributors to COVID-19-associated mortality [11].

Coagulation factor XII (FXII, Hageman factor) is a key mediator of the plasma contact system. Its conversion to activated FXII (FXIIa) initiates multiple cascades affecting coagulation, fibrinolysis, inflammation, including the production of BK through the kallikrein–kinin system (KKS) and complement system [14]. These cascades are involved in pathogen clearance, thrombosis, anaphylactic shock and inflammatory disease [14, 15]. Independent of KKS and complement activation, FXII has been reported to upregulate the expression of pro-inflammatory mediators, such as interleukin (IL)-8, IL-1β, IL-6 and tumour necrosis factor via production of BK [16].

The role of FXII in COVID-19 is not understood; however, indirect evidence, such as excessive fluid accumulation, disseminated intravascular coagulation and observed cytokine storm, suggests that FXII-related pathways may be involved in pathophysiological responses to COVID-19 infection [17]. Endothelial cell permeability during Hantavirus infection involves FXII-dependent activation of the KKS, implicating FXII in the pathophysiology of some viral infections [18]. These physiological effects of FXIIa make evaluating its putative role in COVID-19 disease interesting.

Garadacimab, a fully human immunoglobulin G4 monoclonal antibody, targets FXIIa by binding to the catalytic domain of FXIIa, potently inhibiting the intrinsic coagulation cascade and BK production via inhibition of the KKS [19]. Here we assessed the efficacy and safety of garadacimab versus placebo in patients hospitalised with severe COVID-19.

## Methods

### Study Design and Participants

This was a prospective, phase 2, multicentre, randomised, double-blind, placebo-controlled, parallel-group study across 14 sites conducted in the USA between 1 July 2020 and 12 January 2021 (Online Resource 2). Patients received standard-of-care (SOC) treatment and were randomised (1:1) to receive either a single intravenous dose of garadacimab (700 mg) or matching placebo. The study consisted of a screening period of ≤ 2 days and a 28-day treatment period. SOC referred to all drugs starting on or after administration of study treatment and before Day 28, was permitted throughout the study, including anti-IL-6/anti-IL-6R or investigational products with emergency-use authorisation only. Concomitant therapy was defined as medication starting before and maintained during the study.

Key inclusion criteria included: ≥ 18 years of age; positive SARS-CoV-2 infection confirmed via polymerase chain reaction test within 14 days before screening; interstitial pneumonia confirmed on chest computed tomography or X-ray; and presence of severe COVID-19 disease 24 h before screening. Key exclusion criteria included: requirement for intubation and mechanical ventilation at time of randomisation; presence of comorbid conditions before randomisation and before SARS-CoV-2 infection; active bleeding or clinically significant coagulopathy or clinically significant risk of bleeding; and a history of venous thrombosis or prothrombotic disorder ≤ 3 months before study enrolment. Patients with known hypersensitivity to garadacimab or any excipients of garadacimab [20] were also excluded from the study. See Online Resource 1 for details of the full inclusion and exclusion criteria.

The study was approved by independent ethics committees/institutional review boards of the participating study sites and was conducted in accordance with International Council for Harmonisation Good Clinical Practice Guideline and provisions of the Declaration of Helsinki. Written consent was provided by all patients or by a legally authorised representative on the patient’s behalf.

### Outcomes

Primary efficacy endpoint was the incidence of progression to tracheal intubation (TI) or death before TI from randomisation to Day 28 with garadacimab compared with placebo. Secondary efficacy endpoints included all-cause mortality, incidence of TI from randomisation to Day 28, clinical status as assessed on an 8-point National Institute of Allergy and Infectious Diseases (NIAID) ordinal scale, use of continuous positive airway pressure (CPAP) or bi-level positive airway pressure (BiPAP), use of high-flow nasal cannula (HFNC), median change in Sequential Organ Failure Assessment (SOFA) score and median hospital length of stay. The pharmacokinetics of garadacimab was also assessed with full details to be presented elsewhere.

In this study, the number and percentage of patients who experienced an adverse event (AE) on or after administration of garadacimab or placebo [i.e. a treatment-emergent AE (TEAE)] was assessed. TEAEs, serious TEAEs and AEs of special interest (AESI: abnormal bleeding events, thromboembolic events and severe hypersensitivity, including anaphylaxis) were reported. AEs were coded using the Medical Dictionary for Regulatory Activities version 21.1 (or higher).

### Pharmacodynamics

Blood samples were collected before dosing and at 30 min and 6 h after dosing on Day 1, and Days 2, 7, 14, 21 and 28. Samples were analysed from the safety analysis population by the central laboratory using validated methods.

For both the garadacimab and placebo groups pharmacodynamic biomarkers (activated partial thromboplastin time [aPTT], FXII levels and FXIIa-mediated kallikrein activity [FXIIa-mKA] measured to assess target activation), were evaluated as exploratory biomarkers. Further coagulation biomarkers including prothrombin time/international normalised ratio and D-dimer were also assessed.

### Statistical Analysis

Comparisons of the two study groups for the primary efficacy analysis—and all-cause mortality, incidence of TI and proportion of patients using BiPAP/CPAP—were assessed by Firth logistic regression model including treatment group, age group as a continuous covariate, gender (male or female) and baseline comorbidities (yes or no) as categorical covariates. Comorbidities included hypertension, diabetes and obesity [defined as body mass index (BMI) ≥ 30 kg/m^2^]. A two-sided *p*-value was estimated from the model. The proportion difference and associated 95% confidence interval (CI) were estimated using the method described by Ge et al. [21].

NIAID ordinal scale frequency and proportion of patients with an improvement from baseline of ≥ 2 points were summarised using descriptive statistics. Hospital length of stay was analysed using a Cox model, including treatment group, gender, age as a continuous covariate and baseline comorbidities as categorical covariates. Hazard ratios, 95% CIs and 2-sided Wald *p*-values for hypothesis testing were estimated from the model.

Efficacy outcomes were assessed in the intention-to-treat (ITT) population, comprising all screened patients with randomisation numbers who were assigned to treatment. The ITT population was analysed according to the treatment to which patients were randomised, regardless of the treatment they received. Safety was assessed according to the treatment each patient received, regardless of randomisation. Using a 2-sided α = 0.05 and 1:1 randomisation ratio for garadacimab versus placebo, a total of 124 patients were required to be randomised (garadacimab *n* = 62 vs placebo *n* = 62) to achieve 80% power to detect a treatment difference using a 2-group chi-square test.

## Results

### Patient Characteristics

A total of 131 patients were screened, 124 were randomised and 117 received one dose of study drug (Online Resource 4). All 124 patients were included in the ITT analysis population; the safety analysis population included 117 patients who received ≥ 1 dose of garadacimab (*n* = 58) or placebo (*n* = 59).

Baseline demographics and clinical characteristics were generally balanced between groups and were comparable for age, height, weight and BMI (Table 1). In the ITT analysis, COVID-19 disease characteristics at baseline were similar for patients in the garadacimab and placebo groups (Table 2). Most patients (97.6%) were confirmed as positive for SARS-CoV-2 infection at baseline and 97.6% demonstrated signs of interstitial pneumonia. Many (83.1%) also had ≥ 1 existing comorbidity; 54.8% had hypertension, 38.7% had diabetes and 58.1% were obese (BMI ≥ 30 kg/m^2^) (Table 2).

**Table 1:** Baseline patient demographics and clinical characteristics in the ITT population

	Placebo (*n* = 61)	Garadacimab (*n* = 63)	Total (*N* = 124)
Age (years)			
Mean (± SD)	62.2 (12.74)	62.7 (14.61)	62.5 (13.67)
Age categories, *n* (%)			
< 65 years	33 (54.1)	32 (50.8)	65 (52.4)
18–29 years	0	1 (1.6)	1 (0.8)
30–39 years	4 (6.6)	4 (6.3)	8 (6.5)
40–49 years	6 (9.8)	8 (12.7)	14 (11.3)
50–64 years	23 (37.7)	19 (30.2)	42 (33.9)
≥ 65 years	28 (45.9)	31 (49.2)	59 (47.6)
Sex, *n* (%)			
Male	41 (67.2)	33 (52.4)	74 (59.7)
Female	20 (32.8)	30 (47.6)	50 (40.3)
Ethnicity, *n* (%)			
Hispanic or Latino	10 (16.4)	14 (22.2)	24 (19.4)
Not Hispanic or Latino	48 (78.7)	48 (76.2)	96 (77.4)
Not reported	1 (1.6)	0	1 (0.8)
Unknown	2 (3.3)	1 (1.6)	3 (2.4)
Race, *n* (%)			
American Indian or Alaska Native	0	0	0
Asian	2 (3.3)	2 (3.2)	4 (3.2)
Black or African American	6 (9.8)	7 (11.1)	13 (10.5)
Native Hawaiian or Other Pacific Islander	1 (1.6)	0	1 (0.8)
White	49 (80.3)	44 (69.8)	93 (75.0)
Other	3 (4.9)	8 (12.7)	11 (8.9)
Multiple	0	1 (1.6)	1 (0.8)
Missing	0	1 (1.6)	1 (0.8)
BW at screening, kg			
*n*	59	54	113
Mean (SD)	95.6 (24.65)	97.0 (22.23)	96.2 (23.43)
Height at screening, cm			
*n*	58	56	114
Mean (SD)	170.6 (10.34)	171.3 (9.92)	171.0 (10.10)
BMI at screening, kg/m^2^			
*n*	58	53	111
Mean (SD)	32.78 (7.75)	33.17 (7.23)	32.973 (7.47)
Median (Q1, Q3)	30.86 (27.469, 38.520)	32.00 (28.066, 35.498)	31.74 (27.469, 37.466)
Minimum, maximum	17.31, 54.69	21.34, 55.02	17.31, 55.02

Percentages are calculated with the number of patients (*n*) in each treatment as the denominator

*BMI* body mass index, *BW* body weight, *ITT* intention-to-treat, *Q1* first quartile, *Q3* third quartile, *SD* standard deviation

**Table 2:** COVID-19 disease characteristics at baseline in the ITT population

	Placebo (*n* = 61)	Garadacimab (*n* = 63)	Total (*N* = 124)
Confirmed positive SARS-CoV-2 infection, *n* (%)			
Yes	61 (100)	60 (95.2)	121 (97.6)
No	0	0	0
Missing	0	3	3
Time since onset of symptoms, days^a^			
*n*	61	61	122
Mean (SD)	9.9 (5.40)	9.9 (4.10)	9.9 (4.77)
Median (Q1, Q3)	9.0 (7.0, 13.0)	9.0 (7.0, 12.0)	9.0 (7.0, 13.0)
Minimum, maximum	2, 37	2, 19	2, 37
Time since admission to hospital, days^a^			
*n*	61	61	122
Mean (SD)	2.6 (2.00)	4.4 (11.77)	3.5 (8.46)
Median (Q1, Q3)	2.0 (1.0, 3.0)	2.0 (1.0, 4.0)	2.0 (1.0, 4.0)
Minimum, maximum	1, 10	1, 93	1, 93
Time since admission to ICU, days^a,b^			
*n*	11	8	19
Mean (SD)	2.5 (1.69)	2.3 (1.39)	2.4 (1.54)
Median (Q1, Q3)	2.0 (1.0, 4.0)	2.0 (1.0, 3.5)	2.0 (1.0, 4.0)
Minimum, maximum	1, 6	1, 4	1, 6
Imaging, *n* (%)			
Chest CT	13 (21.3)	14 (22.2)	27 (21.8)
Chest X-ray	59 (96.7)	59 (93.7)	118 (95.2)
Missing	0	2	2
Signs of interstitial pneumonia, n (%)			
Yes	60 (98.4)	61 (96.8)	121 (97.6)
No	1 (1.6)	0	1 (0.8)
Missing	0	2	2
Smoking status, *n* (%)			
Current	2 (3.3)	1 (1.6)	3 (2.4)
Former	16 (26.2)	15 (23.8)	31 (25.0)
Never	42 (68.9)	45 (71.4)	87 (70.2)
Former/never	58 (95.1)	60 (95.2)	118 (95.2)
Missing	1	2	3
Pack-years of cigarettes^c^			
*n*	14	12	26
Mean (SD)	25.2 (25.45)	25.8 (31.40)	25.5 (27.76)
Median (Q1, Q3)	14.5 (6.5, 42.0)	17.1 (1.7, 36.1)	16.1 (6.2, 41.3)
Minimum, Maximum	1, 87	0, 112	0, 112
Presence and type of comorbidity factors, *n* (%)			
Any comorbidity factor	51 (83.6)	52 (82.5)	103 (83.1)
Hypertension	34 (55.7)	34 (54.0)	68 (54.8)
Diabetes	23 (37.7)	25 (39.7)	48 (38.7)
Obesity (BMI ≥ 30 kg/m^2^)	36 (59.0)	36 (57.1)	72 (58.1)
No comorbidity factor	10 (16.4)	11 (17.5)	21 (16.9)
NIAID score			
≥ 2-point improvement at baseline (pre-dose)			
Hospitalised, on invasive mechanical ventilation or ECMO	0	0	–
Hospitalised, on NIV or high-flow O_2_ devices	23 (37.7)	23 (36.5)	–
Hospitalised, requiring supplemental O_2_	34 (55.7)	32 (50.8)	–
Hospitalised, not requiring supplemental O_2_, requiring ongoing medical care	2 (3.3)	3 (4.8)	–
Hospitalised, not requiring supplemental O_2_, no longer requiring medical care	0	0	–
Time point of starting other drugs for COVID-19^d^ *n* (%)			
Prior (ended before randomisation)	0	0	0
Prior and concomitant (started before randomisation, ended after randomisation)	55 (90.2)	50 (79.4)	105 (84.7)
SOC^e^ only (started after randomisation)	44 (72.1)	38 (60.3)	82 (66.1)

*BMI* body mass index, *CT* computed tomography, *ECMO* extracorporeal membrane oxygenation, *ICU* intensive care unit, *ITT* intention-to-treat, *NIAID* National Institute of Allergy and Infectious Diseases, *NIV* non-invasive ventilation, *O*_*2*_ oxygen, *Q1* first quartile, *Q3* third quartile, *SD* standard deviation, *SOC* standard of care

^a^Date (and time) of onset of symptoms/admission to hospital or ICU

^b^Only for patients in ICU at randomisation

^c^Number of cigarettes per day/20 × duration of smoking (years) calculated for current and former smoker

^d^Patients were counted once in each category if anti-COVID-19 drugs had been started

^e^SOC refers to medications starting on or after the administration of study treatment and before Day 28

### Standard of Care and Concomitant Medications

Overall, nearly all patients (90.3%) in the ITT analysis set had received other medications before study enrolment or received SOC with medications other than garadacimab during this study; per protocol SOC was permitted during this study. The percentage of patients receiving SOC starting on or after administration of garadacimab or placebo and before Day 28, was lower with garadacimab (85.7%) than placebo (95.1%). Low molecular weight heparin was amongst the most commonly used SOC medications in all patients (41.1%); use was lower with garadacimab (36.5%) than placebo (45.9%) (Table 3).

**Table 3:** Most commonly used SOC medications in the ITT population during the study

	Placebo, *n* (%) (*n* = 61)	Garadacimab, *n* (%) (*n* = 63)	Total, *n* (%) (*N* = 124)
Any SOC medication	58 (95.1)	54 (85.7)	112 (90.3)
Glucocorticoids	27 (44.3)	25 (39.7)	52 (41.9)
Dexamethasone	18 (29.5)	14 (22.2)	32 (25.8)
Prednisone	5 (8.2)	6 (9.5)	11 (8.9)
Dexamethasone sodium phosphate	4 (6.6)	4 (6.3)	8 (6.5)
Methylprednisolone sodium succinate	4 (6.6)	2 (3.2)	6 (4.8)
Hydrocortisone sodium succinate	3 (4.9)	1 (1.6)	4 (3.2)
Budesonide	2 (3.3)	1 (1.6)	3 (2.4)
Dexamethasone acetate	0	1 (1.6)	1 (0.8)
Hydrocortisone	0	1 (1.6)	1 (0.8)
Methylprednisolone	1 (1.6)	0	1 (0.8)
Heparin group	28 (45.9)	23 (36.5)	51 (41.1)
Enoxaparin	21 (34.4)	18 (28.6)	39 (31.5)
Heparin	8 (13.1)	4 (6.3)	12 (9.7)
Enoxaparin sodium	4 (6.6)	4 (6.3)	8 (6.5)
Heparin sodium	2 (3.3)	0	2 (1.6)
Nucleosides and nucleotides	24 (39.3)	20 (31.7)	44 (35.5)

Percentages are calculated with the number of patients in each treatment as the denominator. SOC was defined as medications starting on or after the administration of study treatment and before Day 28

*ITT* intention-to-treat, *SOC* standard of care

Fewer patients received concomitant medications for COVID-19 with garadacimab (87.3%) than placebo (93.4%) (Online Resource 3). Those administered to > 5% of patients included dexamethasone, remdesivir, dexamethasone sodium phosphate, prednisone and methylprednisolone sodium succinate.

### Primary Efficacy Endpoint

**Fig. 1:**
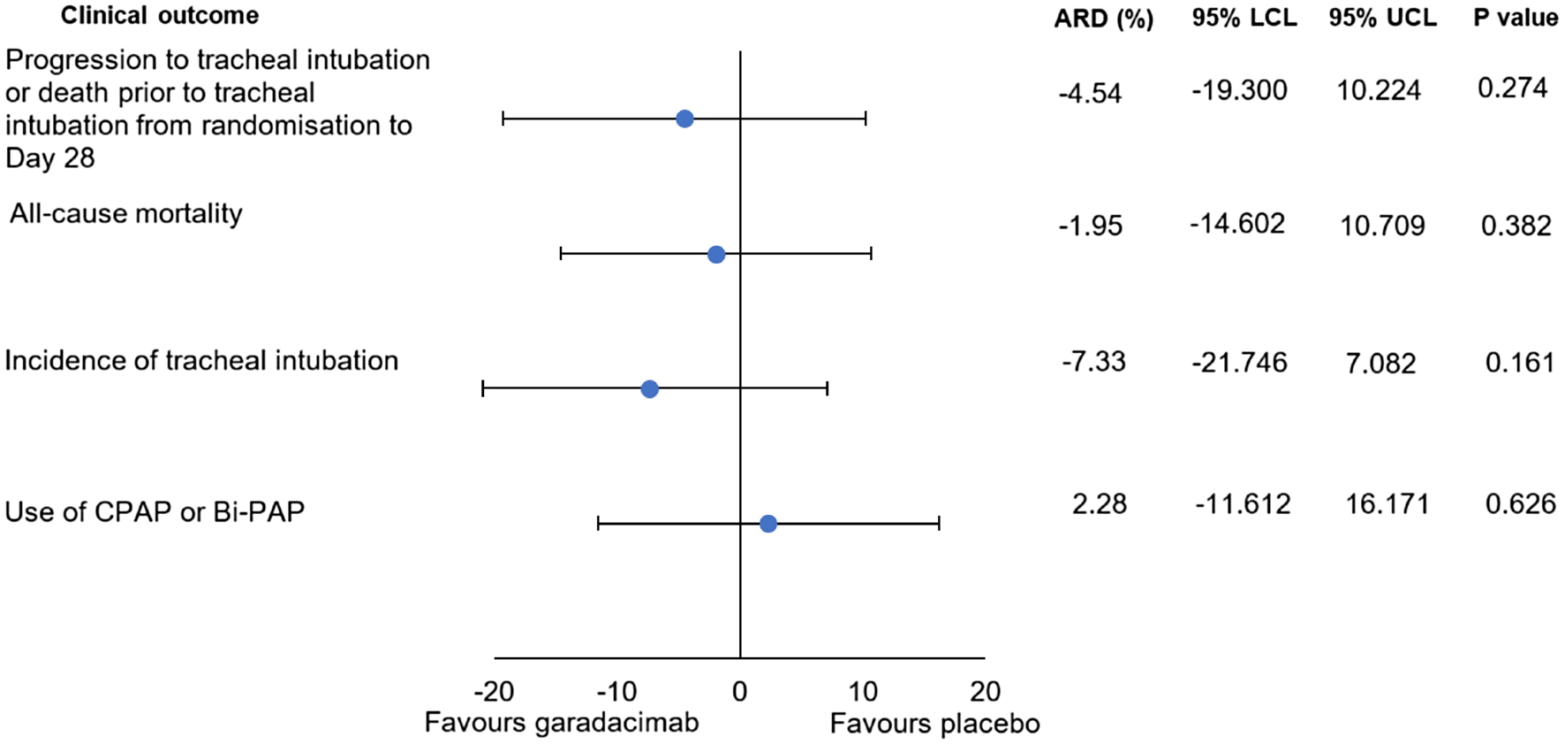
ARD and 95% confidence limits for incidence of clinical outcomes indicative of COVID-19 severity for garadacimab—placebo. Firth logistic regression model includes treatment, age (continuous), sex and baseline comorbidity factors (presence of obesity [BMI ≥ 30 kg/m^2^], diabetes or hypertension) as categorical covariates. Patients with missing endpoint data were assumed not to have experienced the event. *ARD* adjusted risk difference, *Bi-PAP* bi-level positive airway pressure, *BMI* body mass index, *CPAP* continuous positive airway pressure, *LCL* lower 95% confidence limit, *UCL* upper 95% confidence limit

There was no difference in the proportion of patients who progressed to TI or death before TI from randomisation to Day 28 between garadacimab and placebo groups [22.2% vs 26.2%; adjusted risk difference (ARD) 4.54% (95% CI 19.3, 10.2); *p* = 0.274] (Fig. 1).

Subgroup analyses of the primary efficacy endpoint based on age group, sex and baseline comorbidities yielded no meaningful differences between patients receiving garadacimab and placebo.

### Secondary Outcomes

#### Incidence of All-cause Mortality and Tracheal Intubation

Incidence of all-cause mortality and TI was similar in the two groups (Fig. 1). All-cause mortality: garadacimab 17.5% versus placebo 18.0% [ARD 1.95% (95% CI 14.6, 10.7); *p* = 0.382]; TI: garadacimab 17.5% versus placebo 24.6% [ARD 7.33% (95% CI 21.7, 7.1); *p* = 0.161].

#### Clinical Status Assessed by NIAID Scale

The percentage of patients with ≥ 2-point improvement in NIAID score at Day 28 compared with baseline, remained lower with garadacimab (66.7%) than placebo (72.1%) (Table 4).

**Table 4:** Clinical status for the ITT population (*N* = 124) as assessed on the 8-point NIAID ordinal scale

NIAID category (NIAID score), *n* (%)	Placebo (*n* = 61)	Garadacimab (*n* = 63)
≥ 2-point improvement at any time compared with baseline clinical status at Day 28 (EOS)	44 (72.1)	42 (66.7)
≥ 2-point improvement compared with baseline	39 (63.9)	36 (57.1)
1. Death	11 (18.0)	11 (17.5)
2. Hospitalised, on invasive mechanical ventilation or ECMO	2 (3.3)	1 (1.6)
3. Hospitalised, on NIV or high-flow O_2_ devices	2 (3.3)	0
4. Hospitalised, requiring supplemental O_2_	1 (1.6)	2 (3.2)
5. Hospitalised, not requiring supplemental O_2_, requiring ongoing medical care	0	0
6. Hospitalised, not requiring supplemental O_2_, no longer requiring medical care	0	0
7. Not hospitalised, limitation on activities and/or requiring home O_2_	14.0 (23.0)	11.0 (17.5)
8. Not hospitalised, no limitations on activities	25 (41.0)	26 (41.3)
Not performed	5 (8.2)	6 (9.5)
Missing	1 (1.6)	6 (9.5)

≥ 2-point improvement in NIAID score compared with baseline

NIAID scores are reported at Day 28 (final day of assessment)

*ECMO* extracorporeal membrane oxygenation, *EOS* end of study, *ITT* intention-to-treat, *NIAID* National Institute of Allergy and Infectious Diseases, *NIV* non-invasive ventilation, *O*_*2*_ oxygen

#### Use of BiPAP/CPAP and Incidence of HFNC

There was no difference between the use of BiPAP/CPAP (Fig. 1) and incidence of HFNC (data not shown) between the two groups. BiPAP/CPAP use: garadacimab 19.0% versus placebo 16.4% [ARD 2.28% (95% CI 11.6, 16.2); *p* = 0.626]. Incidence of HFNC: garadacimab 14.3% versus placebo 18.0% [ARD 2.04% (95% CI 15.3, 11.3); *p* = 0.382].

#### Length of Hospital Stay

There was no difference in the mean length of hospital stay between garadacimab and placebo groups [hazard ratio 1.17 (95% CI 0.768, 1.783); *p* = 0.767; data not shown].

### Pharmacodynamics

#### Coagulation Biomarkers

**Fig. 2:**
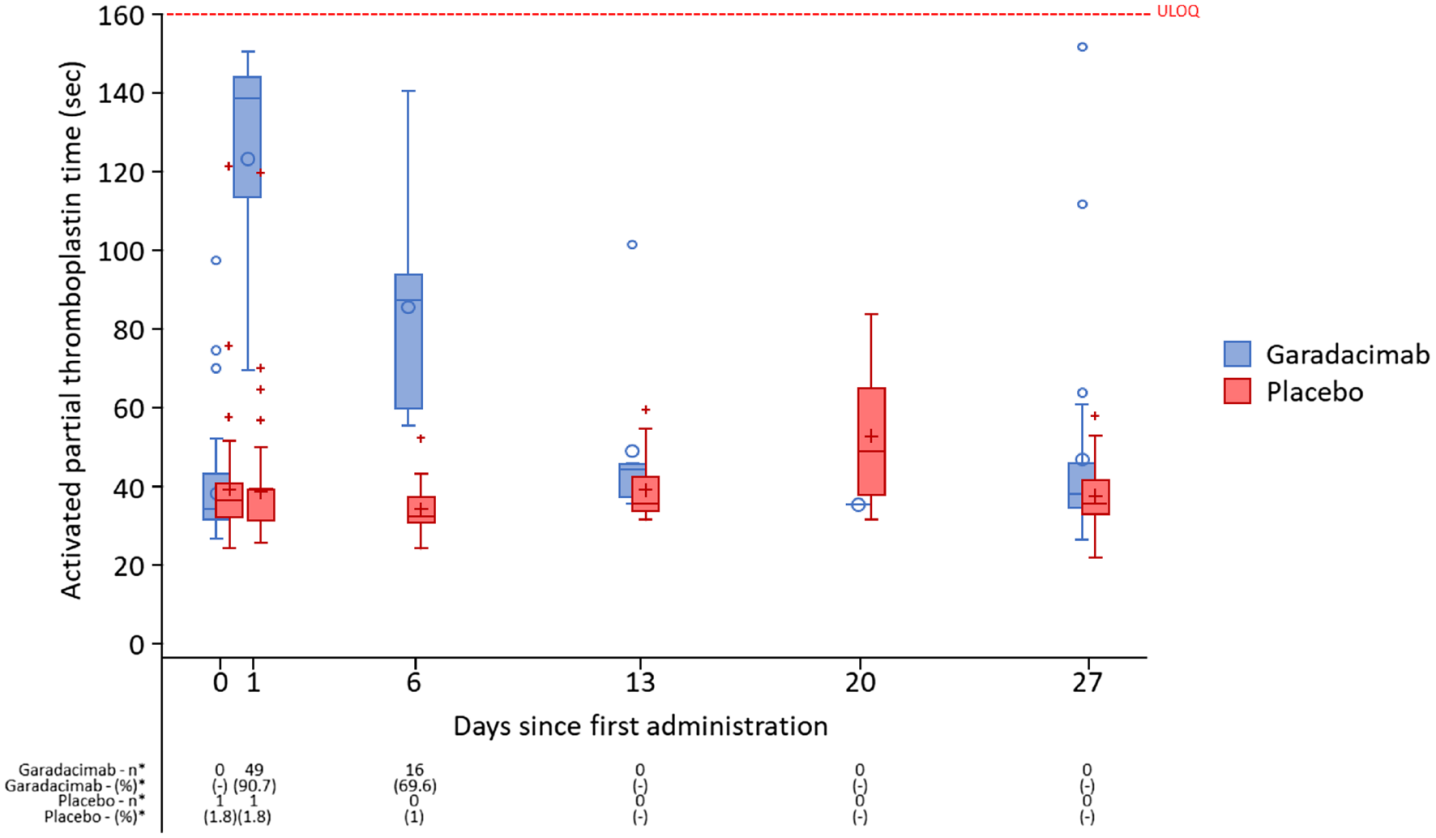
Boxplots of aPTT in the ITT population. *n (%) shows the number (%) of subjects who had aPTT values above the ULOQ in the respective treatment group at the given visit. *aPTT* activated partial thromboplastin time, *ITT* intention-to-treat, *ULOQ* Upper limit of quantification

Target engagement of garadacimab was shown by an increase and prolongation of aPTT to Day 14 (Fig. 2). FXII levels transiently increased after administration of garadacimab versus placebo to Day 14, whilst FXIIa-mKA was suppressed to Day 28. Despite elevated aPTT levels, there were no perturbations in other coagulation biomarkers (D-dimer, prothrombin time/international normalised ratio) with garadacimab versus placebo (data not shown).

### Treatment-Emergent Adverse Events

Nearly two-thirds of patients (64.1%) experienced ≥ 1 TEAE (Table 5). Garadacimab was associated with fewer TEAEs (60.3%) than placebo (67.8%). Most TEAEs were mild or moderate in severity in both groups (Table 5). Garadacimab was associated with fewer serious TEAEs compared with placebo [30 vs 45 events (Table 5)] and the most frequently reported serious TEAEs in both groups involved respiratory, vascular and cardiac disorders, and infections (Table 6).

**Table 5:** Summary of TEAEs reported in the safety population

	Placebo (*n* = 59)		Garadacimab (*n* = 58)		Total (*N* = 117)	
	*n* (%)	E	*n* (%)	E	*n* (%)	E
Any TEAE	40 (67.8)	172	35 (60.3)	131	75 (64.1)	303
Treatment-related	2 (3.4)	2	2 (3.4)	3	4 (3.4)	5
TEAE occurring ≤ 24 h after administration	10 (16.9)	15	10 (17.2)	16	20 (17.1)	31
TEAEs leading to study discontinuation	0	–	1 (1.7)	1	1 (0.9)	1
TEAEs leading to dose modifications	0	–	0	–	0	–
Treatment-emergent AESIs	6 (10.2)	11	5 (8.6)	8	11 (9.4)	19
Treatment-related	1 (1.7)	1	0	–	1 (0.9)	1
Serious TEAEs	19 (32.2)	45	20 (34.5)	38	39 (33.3)	83
Treatment-related	1 (1.7)	1	0	–	1 (0.9)	1
Serious TEAEs occurring ≤ 24 h after administration	1 (1.7)	3	1 (1.7)	2	2 (1.7)	5
Fatal TEAEs	11 (18.6)	11	12 (20.7)	12	23 (19.7)	23
Treatment-related	0	–	0	–	0	–
TEAEs by intensity						
Mild	30 (50.8)	76	27 (46.6)	61	57 (48.7)	137
Moderate	17 (28.8)	51	13 (22.4)	40	30 (25.6)	91
Severe	19 (32.2)	45	17 (29.3)	30	36 (30.8)	75

*AESI* adverse event of special interest, *SAE* serious adverse event, *TEAE* treatment-emergent adverse event

**Table 6:** Serious adverse events reported in the safety population

Serious TEAE	Placebo (*n* = 59)		Garadacimab (*n* = 58)		Total (*N* = 117)	
	*n* (%)	E	*n* (%)	E	*n* (%)	E
Respiratory, thoracic and mediastinal disorders	12 (20.3)	15	15 (25.9)	20	27 (23.1)	35
Respiratory failure	7 (11.9)	8	6 (10.3)	6	13 (11.1)	14
Hypoxia	1 (1.7)	1	4 (6.9)	5	5 (4.3)	6
Acute respiratory failure	1 (1.7)	1	4 (6.9)	4	5 (4.3)	5
Pulmonary embolism	2 (3.4)	2	2 (3.4)	3	4 (3.4)	5
Pneumothorax	1 (1.7)	1	1 (1.7)	1	2 (1.7)	2
Acute respiratory distress syndrome	1 (1.7)	1	0	–	1 (0.9)	1
Dyspnoea	1 (1.7)	1	0	–	1 (0.9)	1
Epistaxis	0	–	1 (1.7)	1	1 (0.9)	1
Vascular disorders	5 (8.5)	7	4 (6.9)	5	9 (7.7)	12
Deep vein thrombosis	1 (1.7)	1	3 (5.2)	3	4 (3.4)	4
Hypotension	3 (5.1)	3	1 (1.7)	1	4 (3.4)	4
Arterial thrombosis	1 (1.7)	1	0	–	1 (0.9)	1
Peripheral artery thrombosis	1 (1.7)	1	0	–	1 (0.9)	1
Peripheral ischaemia	0	–	1 (1.7)	1	1 (0.9)	1
Venous thrombosis	1 (1.7)	1	0	–	1 (0.9)	1
Infections and infestations	6 (10.2)	11	2 (3.4)	4	8 (6.8)	15
Septic shock	4 (6.8)	4	1 (1.7)	1	5 (4.3)	5
Pneumonia	1 (1.7)	1	1 (1.7)	2	2 (1.7)	3
Sepsis	2 (3.4)	2	0	–	2 (1.7)	2
Diverticulitis	0	–	1 (1.7)	1	1 (0.9)	1
Enterobacter bacteraemia	1 (1.7)	1	0	–	1 (0.9)	1
Pneumonia *Escherichia*	1 (1.7)	1	0	–	1 (0.9)	1
Pneumonia streptococcal	1 (1.7)	1	0	–	1 (0.9)	1
Cardiac disorders	1 (1.7)	1	5 (8.6)	5	6 (5.1)	6
Cardiac arrest	1 (1.7)	1	3 (5.2)	3	4 (3.4)	4
Sinus tachycardia	0	–	1 (1.7)	1	1 (0.9)	1
Tachycardia	0	–	1 (1.7)	1	1 (0.9)	1
Nervous system disorders	2 (3.4)	4	1 (1.7)	1	3 (2.6)	5
Brain hypoxia	0	–	1 (1.7)	1	1 (0.9)	1
Cerebellar infarction	1 (1.7)	1	0	–	1 (0.9)	1
Cerebral infarction	1 (1.7)	1	0	–	1 (0.9)	1
Haemorrhagic stroke	1 (1.7)	1	0	–	1 (0.9)	1
Subarachnoid haemorrhage	1 (1.7)	1	0	–	1 (0.9)	1
Blood and lymphatic system disorders	2 (3.4)	2	1 (1.7)	1	3 (2.6)	3
Anaemia	1 (1.7)	1	0	–	1 (0.9)	1
Disseminated intravascular coagulation	0	–	1 (1.7)	1	1 (0.9)	1
Thrombocytopenia	1 (1.7)	1	0	–	1 (0.9)	1
Renal and urinary disorders	2 (3.4)	2	1 (1.7)	1	3 (2.6)	3
Acute kidney injury	2 (3.4)	2	1 (1.7)	1	3 (2.6)	3
General disorders and administration site conditions	1 (1.7)	1	0	–	1 (0.9)	1
Asthenia	1 (1.7)	1	0	–	1 (0.9)	1
Injury, poisoning and procedural complications	1 (1.7)	1	0	–	1 (0.9)	1
Subdural haematoma	1 (1.7)	1	0	–	1 (0.9)	1
Metabolism and nutrition disorders	1 (1.7)	1	0	–	1 (0.9)	1
Hypoglycaemia	1 (1.7)	1	0	–	1 (0.9)	1
Psychiatric disorders	0	–	1 (1.7)	1	1 (0.9)	1
Mental status changes	0	–	1 (1.7)	1	1 (0.9)	1

Percentages were calculated with the number of patients in each treatment group as the denominator. AEs were coded using MedDRA version 23.1

*AE* adverse event, *E* event, *MedDRA* Medical Dictionary for Regulatory Activities, *TEAE* treatment-emergent adverse event

Eleven patients experienced a total of 19 AESIs: 10 patients from the garadacimab group had 15 thromboembolic events not related to garadacimab and one placebo recipient had four abnormal bleeding events. No garadacimab-related bleeding events were reported, despite permitted anticoagulant coadministration. All AESIs, except one suspected unexpected serious adverse reaction (in the placebo group), were considered not related to the investigational product. Overall, 19.7% patients had fatal TEAEs and the amount was similar between groups. No deaths were considered treatment related and no safety concerns or signals emerged from this study.

One patient receiving garadacimab, discontinued treatment because of an SAE (cardiac arrest), with a fatal outcome assessed as not related to garadacimab.

## Discussion

In this phase 2 study of garadacimab in patients hospitalised with severe COVID-19, the primary efficacy endpoint, incidence of TI or death before intubation from randomisation to Day 28, was not met. Although there were no differences between the two groups in rate of TI or death before intubation, there were small numerical differences in favour of garadacimab. The heterogeneity of the study population and small sample size may account for the lack of a clear differentiation between the groups. Subgroup analyses did not reveal any impact of age group, sex or baseline comorbidities on the primary efficacy endpoint.

The safety profile for garadacimab in this severely ill patient population was benign, with no garadacimab-related deaths, thromboembolic events or bleeding events—an important observation as many participants study were receiving anticoagulation therapy.

The high incidence of thromboembolic events in patients with severe disease was expected given that COVID-19 activates the coagulation system thereby propagating a prothrombotic state [22]. Patients with severe COVID-19 are reportedly more susceptible to bleeding events when receiving anticoagulation therapy than those with mild disease [23]. Prolonged aPTT seen in this study, indicated FXII inhibition by garadacimab in patients with severe COVID-19. Prothrombin time/international normalised ratio was unaffected by the administration of garadacimab, as observed in previous studies, consistent with the inhibition of the intrinsic but not extrinsic coagulation pathways [24]. The lack of bleeding events observed with garadacimab in our study is consistent with the observation that patients who have a congenital FXII deficiency do not exhibit a bleeding phenotype, despite demonstrating prolonged aPTT [25].

In our study, anticoagulant therapy was initiated in approximately half of patients during the 28-day study period. Even with coadministration of heparin in approximately one-third of patients receiving garadacimab, there were no differences in abnormal bleeding events between the two groups. In a single-centre study conducted in the USA from March to May 2020, only 4% of patients hospitalised with COVID-19 disease of any severity did not receive anticoagulants in either therapeutic, prophylactic or subclinical doses [26]. Variations in regional practices in the treatment of COVID-19, particularly early in the pandemic, may account for the differences in provisions for anticoagulant therapy between this and the single-centre study [26]. Furthermore, the effect of COVID-19 on thrombosis was becoming increasingly known, necessitating the need for anticoagulation therapy. Observations of benefits/risk of prophylactic therapy compared with therapeutic anticoagulation therapy was not possible in this study.

The benign safety findings in this study are consistent with those reported for garadacimab in healthy volunteers [24] and patients with hereditary angioedema (HAE) [27]. In patients with HAE, all TEAEs were mild with no serious TEAEs [27]. Most (77%) TEAEs were assessed by investigators as unrelated to treatment, and all those related to garadacimab resolved over time with no requirement for concomitant medication or study discontinuation [27].

Limitations to this study include the challenges associated with conducting a study during an active pandemic, which necessitated the use of a flexible protocol allowing the use of any treatment that may have proved therapeutically useful. Patients often received numerous concomitant therapies alongside garadacimab, thus limiting the ability to separate the use of therapeutic and prophylactic anticoagulant doses, resulting in the lack of efficacy regarding primary endpoints. Further, the heterogeneity of the study population and small sample size may account for the lack of clear differentiation between the groups.

## Conclusion

In patients with severe COVID-19, garadacimab did not confer a clinical benefit over placebo. Transient aPTT prolongation and suppressed FXIIa-mKA showed target engagement of garadacimab with no associated bleeding events, even with co-administered anticoagulation therapy. The safety profile of garadacimab was benign, consistent with previous studies in healthy volunteers and patients with HAE. These promising safety findings provide important supporting evidence for the ongoing clinical development of garadacimab in other diseases.

## Supplementary Information

Below is the link to the electronic supplementary material.

Supplementary file1 (PDF 122 KB)
