# Supplementary material for: Efficacy and Safety of Garadacimab in Combination with Standard of Care Treatment in Patients with Severe COVID-19
Source: Lung. 2023 Mar 31;201(2):159–70. doi: 10.1007/s00408-023-00615-9 (PMC10064633; doi:10.1007/s00408-023-00615-9)
Supplement: Supplementary file 1 — Supplementary file1 (PDF 122 KB) [file 408_2023_615_MOESM1_ESM.pdf]

ONLINE RESOURCE

From Papi A et al. Efficacy and Safety of Garadacimab in Combination with Standard of Care Treatment in Patients with Severe COVID-19. Lung.

Corresponding author: Alberto Papi (Alberto.papi@unife.it)

Online Resource 1: Study participants

Inclusion criteria

1. Capable of providing written informed consent. If necessary, an individual legally permitted to make medical decisions on the subject's behalf could have provided written informed consent
2. Willing and able to adhere to all protocol requirements
3. Aged ≥18 years at the time that informed consent was obtained
4. Positive for SARS-CoV-2 infection as determined using a molecular diagnostic test (reverse transcription polymerase chain reaction or equivalent) approved by regulatory authorities (including US Food and Drug Administration or Brazilian Health Regulatory Agency) or allowed under an emergency-use authorisation within 14 days before screening. If a false-negative result was suspected, the SARS-CoV-2 test was repeated during the screening period
5. Chest computed tomography scan or X-ray results confirming interstitial pneumonia
6. Severe COVID-19 disease as evidenced by ≥1 of the following criteria at screening, including within 24 hours before screening:
    - Respiratory frequency >30 breaths per minute
    - Saturation of peripheral (capillary) oxygen ≤93% on room air
    - Ratio of partial pressure of arterial oxygen to fraction of inspired oxygen (PaO_2/FiO_2 ratio) <300
    - Ratio of arterial oxygen saturation to fraction of inspired oxygen (SaO_2/FiO_2 ratio) <218 (if PaO_2/FiO_2 ratio was not available)
    - Radiographic lung infiltrates >50%

Exclusion criteria

1. Currently enrolled, planning to enrol or participated, within the past 30 days, in a clinical study requiring administration of an investigational product, including expanded access or compassionate use with the only exception being the administration of convalescent plasma. Administration of an investigational product is permitted only if an emergency-use authorisation has been granted (e.g. remdesivir). Additionally, off-label use of approved drugs (e.g. anti-interleukin [IL]-6/anti-IL-6R) is also permitted
2. Females who were pregnant/breastfeeding
3. Intubated and requiring mechanical ventilation (including extracorporeal membrane oxygenation) at the time of randomisation

4. Expectation for intubation within the first 24 hours after administration of either garadacimab or placebo
5. Active do-not-intubate or do-not-resuscitate order
6. Expected survival of <48 hours
7. Presence of any of the following comorbid conditions before randomisation and before SARS-CoV-2 infection:
    - Severe heart failure (New York Heart Association Class IV)
    - End-stage renal disease (Stage ≥4) or need for renal replacement therapy
    - Biopsy-confirmed cirrhosis, portal hypertension or hepatic encephalopathy
    - Malignancy (Stage IV)
    - Chronic lung disease requiring the use of oxygen at home
    - Active tuberculosis disease
8. Active bleeding or clinically significant coagulopathy (e.g. international normalised ratio >1.5) or clinically significant risk of bleeding (e.g. recent intracranial haemorrhage or bleeding peptic ulcer within the past 4 weeks)
9. History of venous thrombosis, myocardial infarction or cerebrovascular event within the 3 months before enrolment, or a prothrombotic disorder (e.g. antithrombin III, protein C or protein S deficiency)
10. Patients with known or suspected Grade 3 or 4 infusion-related reaction or hypersensitivity (per the National Cancer Institute Common Terminology Criteria for Adverse Events) to monoclonal antibody therapy
11. Currently receiving therapy not permitted during the study
12. Female subject of childbearing potential or fertile male patients not using or not willing to use an acceptable method of contraception for 90 days after treatment
13. Any clinical or laboratory abnormality or other underlying conditions (e.g. psychological disorders, substance abuse) that would have rendered the subject unsuitable for participation in the study, in the opinion of the investigator

Online Resource 2: Study sites

Supplementary Table. Location of the 14 study sites across the USA.

Name of centre	State
Nova Clinical Research, LLC	Bradenton, FL
Theia Clinical Research, LLC	Saint Petersburg, FL
MercyOne North Iowa Medical Center	Mason City, IA
Northeast Iowa Medical Education Foundation	Waterloo, IA
Lahey Hospital and Medical Center	Burlington, MA
University of Mississippi Medical Center	Jackson, MS
Holy Name Hospital	Teaneck, NJ
Inspira Health Center Vineland	Vineland, NJ
Sisters of Charity Hospital/ St. Joseph's Campus	Buffalo, NY
Carolina Institute for Clinical Research	Fayetteville, NC
Monument Health Clinical Research	Rapid City, SD
PharmaTex Research	Amarillo, TX
UT Health Science Center, McGovern Medical School	Houston, TX
Inova Alexandria Hospital	Alexandria, VA

Online Resource 3

Supplementary Table. Other drugs for COVID-19 during the study in the ITT population.

Drug, *n* (%)	Placebo (*n*=61)	Garadacimab (*n*=63)	Total (*N*=124)
**Any drug used to treat COVID-19**	57 (93.4)	55 (87.3)	112 (90.3)
**Corticosteroids, weak (Group 1)**	1 (1.6)	0	1 (0.8)
Hydrocortisone	1 (1.6)	0	1 (0.8)
**Glucocorticoids**	56 (91.8)	55 (87.3)	111 (89.5)
Dexamethasone	51 (83.6)	44 (69.8	95 (76.6)
Dexamethasone sodium phosphate	7 (11.5)	11 (17.5)	18 (14.5)
Prednisone	6 (9.8)	7 (11.1)	13 (10.5)
Methylprednisolone sodium succinate	4 (6.6)	4 (6.3)	8 (6.5)
Hydrocortisone sodium succinate	3 (4.9)	1 (1.6)	4 (3.2)
Dexamethasone acetate	0	1 (1.6)	1 (0.8)
Hydrocortisone	0	1 (1.6)	1 (0.8)
Methylprednisolone	1 (1.6)	0	1 (0.8)
**Interleukin inhibitors**	1 (1.6)	0	1 (0.8)
Tocilizumab	1 (1.6)	0	1 (0.8)
**Neuraminidase inhibitors**	1 (1.6)	0	1 (0.8)
Oseltamivir	1 (1.6)	0	1 (0.8)
**Nucleosides and nucleotides excluding reverse transcriptase inhibitors**	50 (82.0)	42 (66.7)	92 (74.2)
Remdesivir	50 (82.0)	42 (66.7)	92 (74.2)
**Specific immunoglobulins**	2 (3.3)	0	2 (1.6)
Hyperimmune **plasma** COVID-19	2 (3.3)	0	2 (1.6)
**Other immunosuppressants**	0	1 (1.6)	1 (0.8)
Hydroxychloroquine **sul**ph**ate**	0	1 (1.6)	1 (0.8)
**Selective immunosuppressants**	1 (1.6)	0	1 (0.8)
Baricitinib	1 (1.6)	0	1 (0.8)

Percentages are calculated with the number of patients in each treatment as the denominator. Medications are coded using World Health Organisation Drug Dictionary WhoDrug-Global-B3 202003.

ITT, intention-to-treat.

Online Resource 4

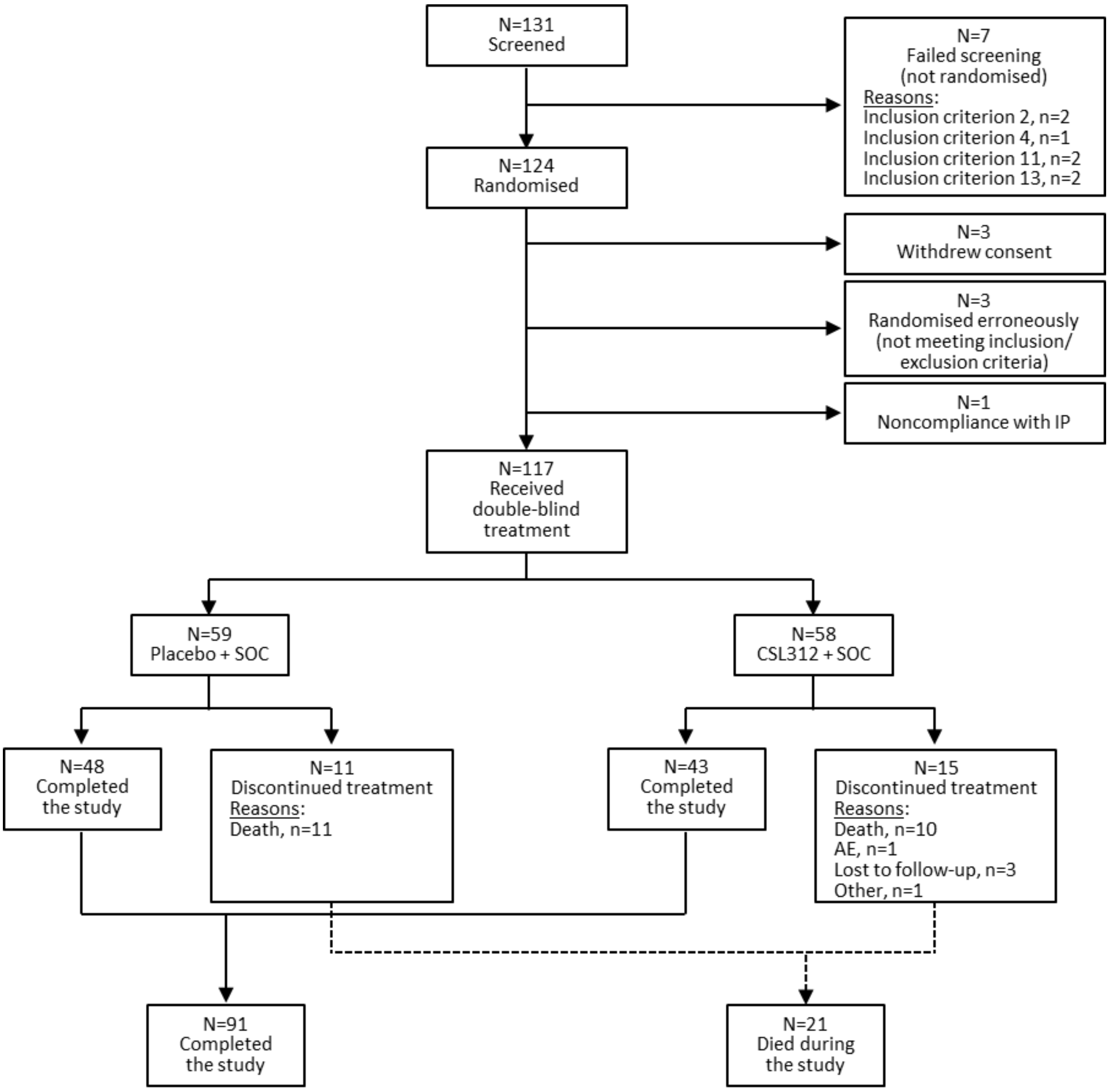

Supplementary Figure. Patient disposition. [a]One patient completed the end-of-study assessment on Day 28 and then died 10 days later. Percentages are based on the number of patients receiving double-blind treatment. 'Discontinued treatment' indicates patients who died, discontinued from the study due to an AE or were lost to follow-up.

AE adverse event; IP, investigational product; SOC, standard of care.

Supplementary References

National Cancer Institute, National Institutes of Health, United States Department of Health and Human Services (DHHS). Common Terminology Criteria for Adverse Events (CTCAE) version 4.0. NIH Publication No. 03-5410. Revised May 2009. Available at: https://evs.nci.nih.gov/ftp1/CTCAE/CTCAE_4.03/Archive/CTCAE_4.0_2009-05-29_QuickReference_8.5x11.pdf. Accessed December 2022.
